# Polarization fatigue of organic ferroelectric capacitors

**DOI:** 10.1038/srep05075

**Published:** 2014-05-27

**Authors:** Dong Zhao, Ilias Katsouras, Mengyuan Li, Kamal Asadi, Junto Tsurumi, Gunnar Glasser, Jun Takeya, Paul W. M. Blom, Dago M. de Leeuw

**Affiliations:** 1 Max-Planck Institute for Polymer Research Ackermannweg 10, 55128 Mainz, Germany; 2 Zernike Institute for Advanced Materials, University of Groningen Nijenborgh 4, 9747 AG, Groningen, The Netherlands; 3 Department of Applied Physics, Osaka University 2-1 Yamadaoka, Suita, 565-0871 Osaka, Japan; 4 Department of Advanced Materials Science, The University of Tokyo 5-1-5 Kashiwanoha, Kashiwa, Chiba, 277-8561, Japan; 5 King Abdulaziz University, Abdullah Sulayman, 22254 Jeddah, Saudi Arabia

## Abstract

The polarization of the ferroelectric polymer P(VDF-TrFE) decreases upon prolonged cycling. Understanding of this fatigue behavior is of great technological importance for the implementation of P(VDF-TrFE) in random-access memories. However, the origin of fatigue is still ambiguous. Here we investigate fatigue in thin-film capacitors by systematically varying the frequency and amplitude of the driving waveform. We show that the fatigue is due to delamination of the top electrode. The origin is accumulation of gases, expelled from the capacitor, under the impermeable top electrode. The gases are formed by electron-induced phase decomposition of P(VDF-TrFE), similar as reported for inorganic ferroelectric materials. When the gas barrier is removed and the waveform is adapted, a fatigue-free ferroelectric capacitor based on P(VDF-TrFE) is realized. The capacitor can be cycled for more than 10^8^ times, approaching the programming cycle endurance of its inorganic ferroelectric counterparts.

Ferroelectric materials possess a spontaneous polarization whose direction may be reversed by applying an external electrical field. The polarization can be used as a Boolean “0” and “1” in ferroelectric random access memories (FeRAM). Here, the binary information is stored in a capacitor and retrieved by applying a switching voltage to obtain a high or a low charge displacement current response, depending on whether the internal polarization was aligned or not with the direction of the applied field. The read-out is therefore destructive. If the polarization direction was changed during the read-out operation then a reset voltage needs to be applied afterwards. During these numerous read and write operations the spontaneous polarization decreases. Although this so-called polarization fatigue has been thoroughly investigated, its origin is still under debate.

Experimental data for the reduction of the spontaneous polarization of inorganic ferroelectric thin films under electrical stress have been reviewed by Tagantsev *et al.* in 2001^1^. The dependence of fatigue on amplitude, frequency and profile of the driving electric field was discussed and models such as domain wall pinning and nucleation inhibition were reviewed. Experimental characteristics and explanations of polarization fatigue in inorganic thin films, bulk ceramics and single crystals have also been reviewed by Lou in 2009^2^. Capacitors consisting of SrBi_2_Ta_2_O_9_ (SBT) exhibit virtually fatigue-free behavior. The endurance is better than 10^12^ cycles^3^. In contrast, traditional Pb(Zr,Ti)O_3_ (PZT) capacitors with Pt electrodes are prone to fatigue. Although the degradation behavior can be improved by using conductive oxide electrodes such as RuO_2_, IrO_2_ and SrRuO_3_^1^, the polarization still decreases after 10^4^ to 10^9^ cycles. The number of cycles at which the polarization starts to decrease is comparable for thin films, bulk ceramics and single crystals^2^, which indicates that a similar degradation mechanism is responsible for the polarization fatigue. Reported models typically comprise two steps^1^: (*i*) electrical stress leads to formation or redistribution of defects and (*ii*) these imperfections influence the spontaneous polarization. For instance, electro-migration of oxygen vacancies can form extended defects capable of pinning domain walls. The oxygen vacancies can also lead to the formation of a dead interface layer at the electrodes. Furthermore, it has been reported that the fatigue of PZT capacitors could be due to local phase decomposition^4^,^5^. Under electrical bipolar stress the ferroelectric PZT perovskite phase is transformed into the paraelectric pyrochlore phase, as confirmed by Micro Raman measurements. Upon annealing the fatigued capacitor in an oxygen ambient, the original ferroelectric perovskite PZT phase was completely restored. Therefore, it was concluded that fatigue is a generic problem of inorganic ferroelectric materials. The origin was argued to be the formation of oxygen vacancies caused by a local, uncompensated high depolarization field.

Contrary to inorganic ferroelectrics, reports on fatigue of organic ferroelectrics are limited. The most studied organic ferroelectric materials are poly(vinylidene-difluoride) (PVDF) and its random copolymer with trifluoroethylene, P(VDF-TrFE). They are investigated due to their potential application in transducers, sensors, actuators and memories. As compared to inorganic ferroelectrics, the remanent polarization is about one order of magnitude lower and the coercive field one order of magnitude higher. However, the advantage of organic ferroelectric materials is compatibility with low temperature flexible substrates and the possibility for up-scaling by large-area solution processing. The remanent polarization and coercive field of ferroelectric capacitors did not change upon bending with a radius of curvature down to 1 cm, which illustrates that organic ferroelectrics are ideal candidates for flexible electronics or system-in-foil applications^6^,^7^. As an example, high performance non-volatile polymer memories on banknotes have recently been realized^8^.

Fatigue in organic ferroelectric capacitors is a major problem as the spontaneous polarization is typically halved already after less than 10^6^ cycles^2^. Fatigue depends on experimental parameters such as temperature, the type of electrodes and the frequency and amplitude of the applied waveform^9^,^10^,^11^. It has been reported for P(VDF-TrFE) that fatigue increases with increasing driving voltage and decreasing frequency. Bipolar driving with either sinusoidal, triangular or rectangular waveforms introduces polarization fatigue, while unipolar switching does not. Application of polymer electrodes, such as poly(3,4-ethylenedioxythiophene) stabilized with polystyrene sulfonic acid (PEDOT:PSS), leads to improved programming cycle endurance^5^. Finally, we note that since ferroelectric polymers are semi-crystalline, fatigue may depend on the degree of crystallinity^12^.

Fatigue in organic ferroelectric materials has been ascribed to charge trapping. Injected charges get trapped at crystalline boundaries and defects, thereby locking the domain walls and reducing the polarization^13^. Increasing the crystallinity concomitantly reduces the number of defects and grain boundaries, resulting in increased reliability. The use of poorly conducting polymer electrodes, or the introduction of an interfacial blocking layer, diminishes charge injection and, hence, fatigue^14^. Apart from the intrinsic domain wall pinning mechanism, fatigue can have an extrinsic origin, such as delamination of the top electrode. A few reports^6^,^10^ mention this delamination and suggest a temperature rise due to the heat dissipation upon continuous cycling as the origin^6^.

Here we systematically investigate fatigue of P(VDF-TrFE) thin-film capacitors. We deliberately varied the frequency and amplitude of the applied waveform. Both unipolar and bipolar switching is considered. We used Au, PEDOT:PSS or PEDOT:PSS covered with Au as top electrode. We show that fatigue is due to delamination of the top electrode. The polarization then decreases proportionally to the decreased electrode area. Thermal and piezoelectric stress is ruled out as the origin. We show that the delamination is due to formation of gases that are expelled from the capacitor. The origin is argued to be electron-induced phase decomposition of the P(VDF-TrFE), similar as reported for the inorganic ferroelectric material PZT. The mechanism is supported by inducing similar damage using high current densities in a scanning electron microscope. We show that when the gas barrier is removed and the waveform is adapted, a fatigue-free ferroelectric capacitor based on P(VDF-TrFE) is realized. The capacitor can be cycled for more than 10^8^ times, approaching the programming cycle endurance of its inorganic ferroelectric counterparts^3^.

## Results and Discussion

### Fatigue of P(VDF-TrFE) capacitors with metal electrodes

**Figure 1:**
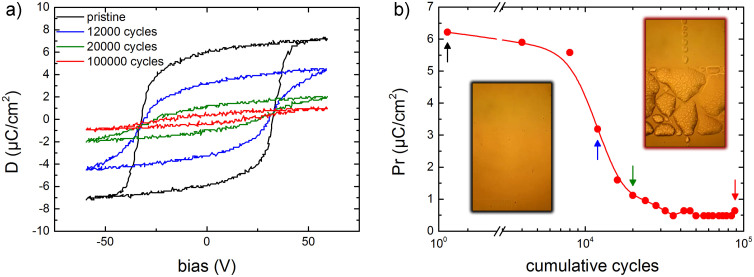
Polarization fatigue. a) Evolution of the displacement loops of a P(VDF-TrFE) ferroelectric capacitor with Au top electrode under continuous cycling. The measurements were performed using a bipolar triangular waveform with a frequency of 1 kHz and an amplitude of 60 V. b) The extracted remanent polarization as a function of the cumulative number of cycles. The arrows indicate the data points corresponding to the displacement loops of Fig. 1a. The inset shows optical micrographs of the pristine and degraded capacitor. The black and red framed micrographs show the top electrode of the pristine and fatigued capacitor, respectively. In the fatigued capacitor the top electrode shows bumps and may have been delaminated.

As an example we present typical fatigue measurements on a ferroelectric P(VDF-TrFE) capacitor in Fig. 1. Au is used as the top electrode. The displacement loops are presented in Fig. 1a as a function of the cumulative number of cycles. The pristine capacitor exhibits a coercive field of 60 MV/m and a remanent polarization of 7 μC/cm^2^, in good agreement with literature values^15^. Upon continuous cycling the coercive field remains constant but the polarization decreases severely. Fig. 1b shows the corresponding remanent polarization as a function of the cumulative number of cycles. The polarization is already halved after about 10^5^ cycles. Comparable numbers have been reported in literature^6^,^16^. The inset shows optical micrographs of the pristine and degraded capacitor. The black framed micrograph shows that the top electrode of the pristine capacitor is smooth, while the red framed micrograph shows that in the fatigued capacitor the top electrode exhibits bumps and may have been delaminated.

**Figure 2:**
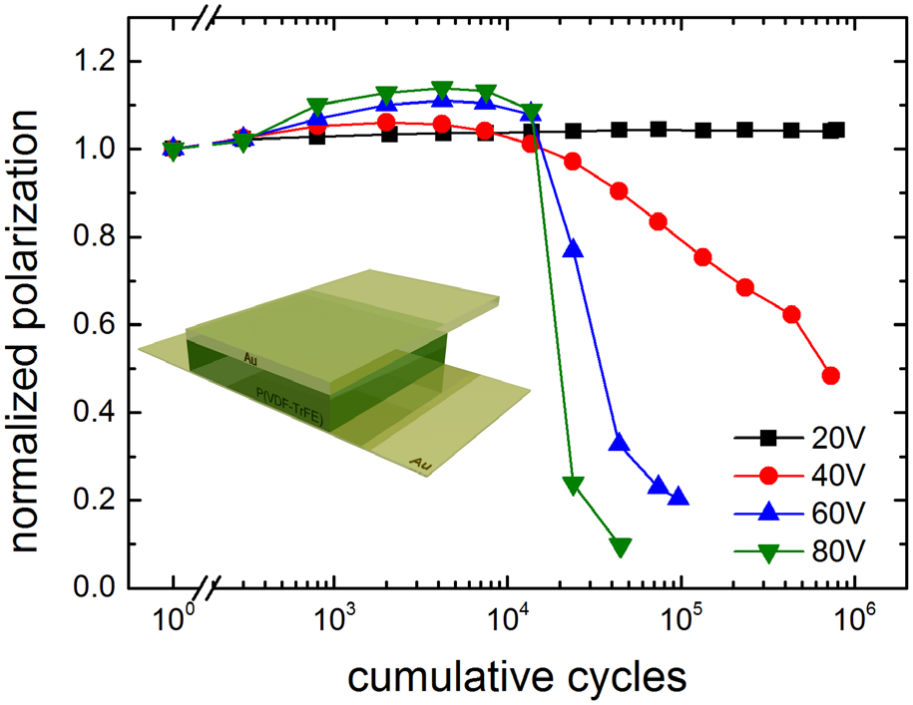
Bias dependence of fatigue. The normalized polarization as a function of the cumulative number of cycles. The frequency was fixed at 100 Hz and the amplitude was varied from 20 V to 80 V. At an amplitude of 20 V the applied electric field is smaller than the coercive field. The data show that the degradation is bias dependent. The onset of the degradation is at about 10^4^ cycles, but the degradation rate strongly increases with increasing bias. The inset depicts the capacitor layout, where a P(VDF-TrFE) thin film is sandwiched between two Au electrodes.

To pinpoint the origin of the measured fatigue we deliberately varied the amplitude and the frequency of the bipolar triangular waveform. The normalized polarization as a function of the cumulative number of cycles is presented in Fig. 2. The frequency was fixed at 100 Hz and the amplitude was varied from 20 to 80 V. At an amplitude of 20 V the applied electric field is smaller than the coercive field. Hence the ferroelectric polarization does not switch. Not surprisingly, the intermittently measured remanent polarization is constant. Under these measurement conditions the capacitor does not switch and is fatigue-free. As soon as the applied field becomes larger than the coercive field, the ferroelectric material switches, and the polarization decreases with increasing cumulative number of cycles. Fig. 2 shows that the degradation is bias dependent. The onset of degradation is at about 10^4^ cycles, but the fatigue rate strongly increases with increasing bias.

The data show that fatigue only occurs when the polarization is switched. This is in perfect agreement with reported so-called unipolar cycling^9^. The electric field then varies from zero to above the coercive field. The ferroelectric polarization does not switch and the capacitor shows no fatigue. A similar conclusion can be drawn from the frequency dependence, as will be discussed below.

Fig. 2 shows that the polarization initially increases. The polarization enhancement has been observed previously, where it was reported that it might be due to field-induced recrystallization^16^. We note that a similar enhancement can be found, upon close inspection, in other reported data sets, both for organic^17^,^18^ and inorganic ferroelectrics^2^. In all our measurements there is an apparent correlation between the polarization enhancement and the onset of degradation. However, the origin is still elusive.

**Figure 3:**
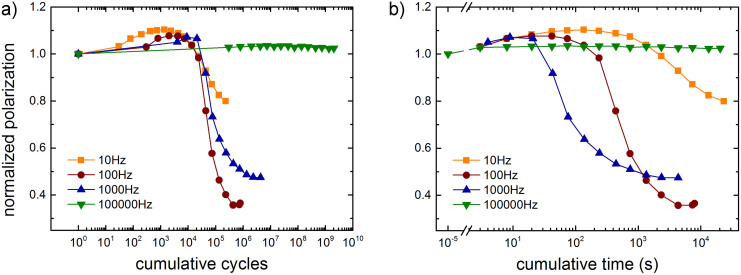
Frequency dependence of fatigue. a) The normalized polarization as a function of the cumulative number of cycles. The amplitude was fixed at 40 V and the frequency was varied from 10 Hz to 100 kHz. At high frequency polarization switching is impeded and therefore the capacitor is fatigue-free. The onset of the degradation is at about 10^4^ cycles. b) Replotted data of normalized polarization as a function of the cumulative time.

The bias dependence suggests that fatigue is related to the switching of the polarization. To substantiate this observation we investigated fatigue as a function of frequency. The frequency was varied from 10 Hz to 100 kHz. The amplitude of 40 V corresponds to an electric field larger than the coercive field. The normalized polarization as a function of the cumulative number of cycles is presented in Fig. 3a. At high frequency, here 100 kHz, the capacitor is fatigue-free. The polarization does not change with the number of cycles. The reason is that at this frequency the ferroelectric does not switch. This is in agreement with the bias dependence, which shows that polarization switching is a prerequisite for fatigue. From the frequency dependence we can further infer that a high electric field alone does not induce fatigue. Fig. 3a shows that fatigue is observed for those frequencies at which the polarization switches. The initial polarization increase is comparable to that of Fig. 2. The onset of degradation is at about 10^4^ cycles. The degradation is almost frequency independent, which suggests that fatigue is dominated by the number of switching events.

The optical micrograph in Fig. 1b shows that delamination of the top Au electrode may occur during the fatigue measurements. When the delamination is due to thermal stress it should depend on the dissipated energy, which is equal to the dissipated power times the cumulative time. Therefore, we replotted the data of Fig. 3a not as a function of the cumulative number of cycles but as a function of cumulative time. As *N* is the number of cycles and *f* is the frequency, *N/f* is the cumulative time. Contrary to previous reports^9^,^19^ that show universal scaling, the data of Fig. 3b do not collapse on a single curve. A clear trend appears. The higher the frequency, the faster the degradation is. We note that the time scales involved are in the order of 10^2^–10^3^ seconds.

In summary, the occurrence of fatigue in a ferroelectric P(VDF-TrFE) capacitor requires polarization switching driven by a bipolar waveform. The fatigue depends on the frequency and amplitude of the applied waveform. These dependencies suggest a power-related problem, which might lead to the observed electrode delamination. However, in the following section we show that thermal stress can be disregarded.

### Thermal analysis

To estimate the temperature rise we approximate the capacitor as a zero-thickness spherical source with constant flux over its area, placed on top of a homogenous semi-infinite substrate. In this case, the thermal resistance is given by: 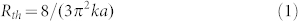
where *a* is the radius of the sphere and *k* is the thermal conductivity of the substrate^20^. The temperature rise, Δ*T*, is then equal to the input power, *P_in_*, multiplied by the thermal resistance: 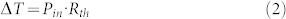
The input power is generated by switching the ferroelectric polarization. The leakage current can be disregarded. We verified that the dissipated energy per cycle is equal to the area in the displacement hysteresis loop. To that end, we recorded the displacement current and we integrated the instantaneous power *I(t)V(t)* over the period of one cycle. The obtained values are equal to the area of the displacement loop for all frequencies at which the ferroelectric switches. We approximate this area by 2*P_r_*·2*V_c_*. The dissipated energy per cycle is then given by: 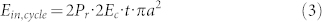
where *P_r_* is the remanent polarization, *E_c_* is the coercive field, *t* the thickness of the ferroelectric layer and *πa*^2^ is the surface area of the capacitor. The total dissipated power is the frequency, *f*, times the energy per cycle. The temperature rise follows from: 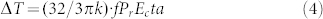
We calculated the temperature rise using a remanent polarization of 7 μC/cm^2^, a coercive field of 60 MV/m, a frequency of 1 kHz and a layer thickness of 500 nm. The dissipated power per unit area is then ~8 kW/m^2^. We take a typical surface area of 1 mm^2^ and for the thermal conductivity we use either 1 W/m·K, a typical value for glass and thermally grown SiO_2_, or 100 W/m·K, a typical value for the highly doped crystalline silicon wafer used as the substrate. The calculated temperature rise is 4°C and 0.04°C respectively, orders of magnitude too low to account for thermal delamination.

To verify these estimations we measured the temperature rise of a segmented polymeric light emitting diode on glass. For an input power of 1 kW/m^2^ we estimated a temperature rise of 1.7°C for a segment with a radius of 2 mm. Here we have assumed that all input power is converted into heat. The estimated value did nicely agree with the experimental value, as measured for the segmented display, of about 2°C.

The time scale for thermal delamination is off by orders of magnitude as well. The time constant, *τ*, is the product of the thermal capacitance of all material that has to be heated and the thermal resistance. 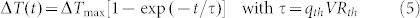
We take a volumetric heat capacity, *q_th_*, of 2 Ws/cm^3^K. The longest time scale is obtained when we assume that the whole substrate, with a volume *V*, has to be heated. The time constant is then at most seconds, still orders of magnitude smaller than the experimental time at which fatigue sets in. We note that the time scale, Eq.(5), does not depend on the dissipated power but only on the substrate properties. Experimentally, however, fatigue rate increases with frequency, hence with increasing dissipated power. Delamination therefore cannot be due to thermal stress. Finally, we used a simplified thermal model. By adapting the thermal resistance, albeit that the values then are unrealistic, a large temperature rise or a long time scale can be calculated. However, these values cannot be obtained simultaneously. A large temperature rise and a long time scale are mutually exclusive. Hence, in summary, fatigue and delamination of the top electrode cannot be due to thermal stress.

### Electrically induced phase decomposition

The modelling of the previous section proved that degradation cannot be due to thermal stress induced by the dissipated power. However, P(VDF-TrFE) is not only ferroelectric but also piezoelectric. The delamination of the top electrode might then be due to the piezoelectric response of the P(VDF-TrFE) layer. Application of an electrical field leads to a contraction in the direction of the field and to a simultaneous expansion in the lateral direction. The resulting lateral strain leads to a mechanical stress at the interface between the top electrode and the P(VDF-TrFE) thin film. The top Au electrode is not compliant, which means that the generated stress cannot be accommodated. Therefore, the top electrode delaminates. The strain, *i.e.* the relative change in the lateral dimension, *l*, is in first order approximation directly proportional to the applied electric field, *Δl/l = d*_31_·*E.* Fig. 2, however, shows that there is no fatigue when the amplitude corresponds to a field below the coercive field. The P(VDF-TrFE) film still exhibits a piezoelectric response though. Therefore, piezoelectricity can be ruled out as the origin of fatigue.

We argue that degradation is due to delamination of the Au top electrode of the fatigued capacitor, as indicated in Fig. 1b. The polarization is then diminished proportionally to the reduced electrode area. The origin of the delamination is gas expelled from the capacitor upon cycling. The gases are formed by phase decomposition of P(VDF-TrFE), induced by the high internal electric fields generated upon switching the polarization.

Gas formation is not unexpected. The radiation chemistry of the homopolymer, PVDF, is well-established and covered in two thorough reviews^21^,^22^. During electron beam irradiation or γ-radiolysis, PVDF undergoes elimination of HF. The following mechanism has emerged. Electrons are injected and trapped at defects, grain boundaries or domain walls. At a given applied external electric field the capacitor is in static equilibrium. All molecular dipoles are compensated for, either internally or by counter-charges at the electrodes. The internal electric field is negligible and the trapped charges remain fixed. However, when the polarization switches temporarily large depolarization fields occur. Hot electrons are injected and trapped electrons are accelerated by this internal electric field. The charge carriers have enough energy to abstract F^−^ ions, which in turn initiate unzipping reactions of the PVDF chains. As a result HF is formed together with unsaturated carbon bonds and cross-linked moieties. The polyene bonds have been identified by optical and infrared absorption measurements, while Raman measurements have confirmed that H^+^ and F^−^ ions are formed in unzipping chain reactions^23^. These reactions only occur when the polarization switches. The gas emission has been monitored using permeable grid electrodes^24^. Gases were predominantly produced at the negatively charged electrode and only during polarization switching. The threshold for gas emission corresponded to the coercive field. Under constant electric field the gas emission decreased by at least an order of magnitude.

**Figure 4:**
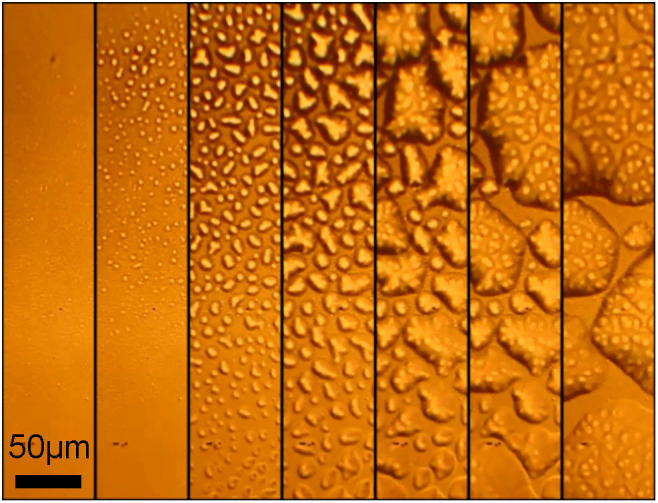
Temporal evolution of the electrode morphology. In-situ optical micrographs on the same spot, taken during a fatigue measurement of a P(VDF-TrFE) capacitor with Au electrodes. The fatigue measurement was performed using a bipolar triangular waveform with a frequency of 1 kHz and an amplitude of 60 V. The left micrograph shows that the Au electrode of the pristine capacitor is smooth. The right micrograph shows that the top electrode is delaminated, which is visible by the naked eye.

To confirm the gas-induced delamination, we looked at the temporal evolution of the electrode morphology. Fig. 4 shows *in-situ* optical micrographs taken during a fatigue measurement. The Au electrode of the pristine capacitor is smooth. During cycling small bumps are formed. With time the number of bumps increases, they grow in size and finally coalesce into macroscopic bubbles. The gold electrode acts as a gas barrier. The gas expelled from P(VDF-TrFE) is accumulated at the interface forming bumps that grow in time. Finally, the top electrode is delaminated, which is visible by the naked eye.

We note that the gas volume required for delamination can be produced by a negligible amount of decomposed polymer. Hence, it is not surprising that the phase decomposition cannot be detected electrically, such as in leakage current or in direct reduction of polarization. Furthermore, we note that for organic ferroelectrics the effect is very pronounced because the electrode is not covalently bound to the polymer layer. The adhesion between the polymer layer and the electrode is limited to van der Waals forces.

Prerequisites for fatigue are therefore both the presence of injected and/or trapped charges and switching of the polarization. This explains why fatigue can be less pronounced when using poorly injecting electrodes and why fatigue only occurs under bipolar switching. Furthermore it might explain the dependence on the waveform. Triangular or sinusoidal pulses yield a similar programming cycle endurance, while the use of rectangular pulses, with abrupt variations in electric field, inevitably leads to an enhanced fatigue behavior. We expect that this mechanism also explains the initial enhancement of the remanent polarization upon cycling. However, we could not pinpoint the direct link. Electrons injected in the pristine polymer might yield extra compensation charges that can stabilize the polarization of parts in the film that had not yet been polarized.

Local phase decomposition has been reported as a generic fatigue mechanism for inorganic ferroelectrics. Optical micrographs of fatigued PZT capacitors showed dark spots due to holes in the Pt top electrode. The delaminated holes are due to evaporation of oxygen and/or Pb/PbO from the interface^4^. Micro Raman measurements, performed in the micron size holes, showed that the ferroelectric perovskite phase was transformed into the paraelectric pyrochlore phase. Upon annealing the degraded capacitor in an O_2_ ambient, the perovskite phase and the accompanying ferroelectric polarization was restored. The phase decomposition was initiated by large depolarization fields that occur when the polarization is switched. It has been argued that the same mechanism holds for other inorganic ferroelectrics such as BaTiO_3_. Here we have shown that it explains fatigue in the organic ferroelectric P(VDF-TrFE).

**Figure 5:**
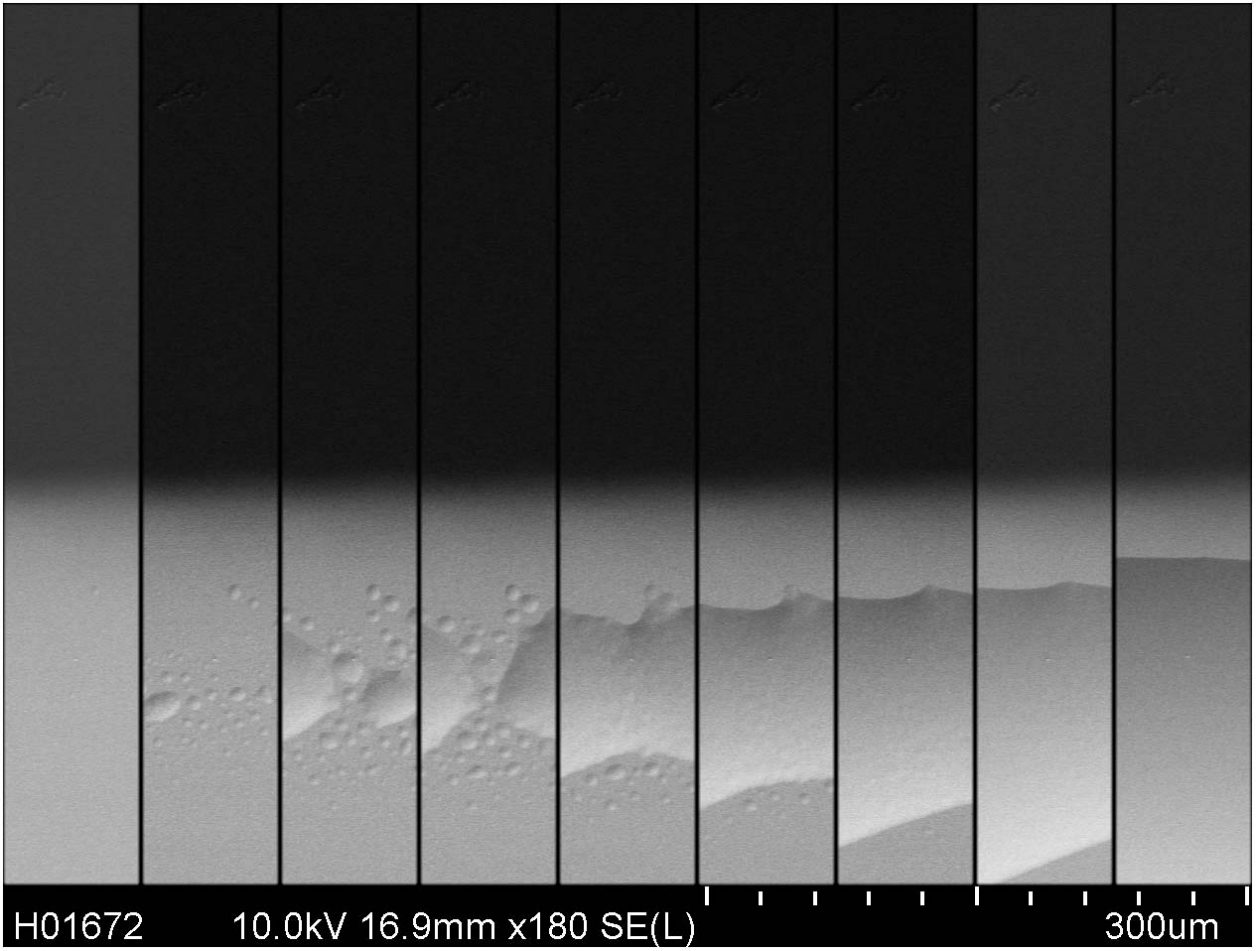
Electron-beam induced damage. A pristine capacitor (left) is exposed to a 10 keV electron beam under analytical SEM conditions with a high probe current of ~1 nA. Real-time images were recorded during the exposure. The composite SEM micrograph shows the time evolution (left to right) of a growing bump on the top Au electrode (light gray area).

The phase decomposition mechanism is confirmed by electron beam-induced damage in a scanning electron microscope. A high probe current was used to expose a pristine capacitor. Real-time images were recorded during the exposure. The composite SEM micrograph of Fig. 5 shows the time evolution (left to right) of a growing bump on the top Au electrode (light gray area).

### Fatigue-free P(VDF-TrFE)-based capacitors

We have shown that the delamination of the top electrode is caused by accumulation of volatile components expelled from the capacitor and blocked by the top electrode. The gases are generated when the polarization switches. The delamination can be avoided by adapting the waveform of the cycling and/or by adapting the layout of the capacitor. The waveform controls the gas generation and diffusion rate. In the adapted layout of the capacitor configuration the gas barrier is removed.

**Figure 6:**
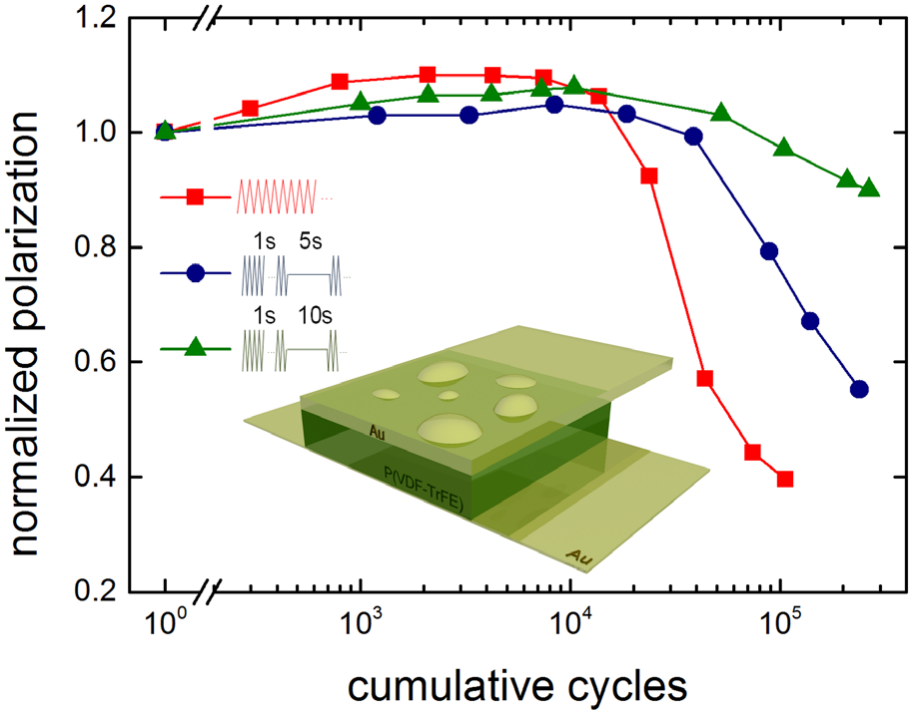
Improved programming cycle endurance. Fatigue measurement of a P(VDF-TrFE) capacitor with Au electrodes. The measurement was performed using a variable duty cycle. The red curve shows the normalized polarization as a function of the cumulative number of cycles for continuous cycling with a bipolar triangular waveform with a frequency of 100 Hz and an amplitude of 40 V. The inset depicts the resulting morphology of the top electrode. The blue and green curves correspond to measurements where the continuous cycling was interrupted every second with a waiting time of 5 and 10 seconds, respectively. The programming cycle endurance increases with decreasing duty cycle.

In Fig. 6 we varied the duty cycle of the waveform. We cycled the capacitor with a triangular waveform of 100 Hz. The red curve shows the normalized polarization as a function of the cumulative number of cycles for continuous cycling. The polarization rapidly decreases and is halved after approximately 5 × 10^4^ cycles. The resulting morphology of the top electrode is schematically depicted in the inset. Next, we adapted the duty cycle. The continuous cycling was interrupted every second with a waiting time of up to 10 seconds, as schematically depicted in the inset of Fig. 6. The cycling endurance increases with waiting time, as shown by the blue (5 s waiting time) and green (10 s waiting time) curves. The measured dependence on duty cycle can be explained as follows. While switching gas is generated by phase decomposition of P(VDF-TrFE). During the waiting time the gas can diffuse out of the capacitor. Gas accumulation at the interface between the P(VDF-TrFE) layer and the top electrode is thereby prevented. The reduced delamination results in improved programming cycle endurance.

To further enhance the endurance and to substantiate the relevance of gas-induced delamination, we varied the capacitor layout. We changed the gold electrode, which is impermeable to gases, to the polymeric conductor PEDOT:PSS, whose gas diffusion coefficient is orders of magnitude higher.

**Figure 7:**
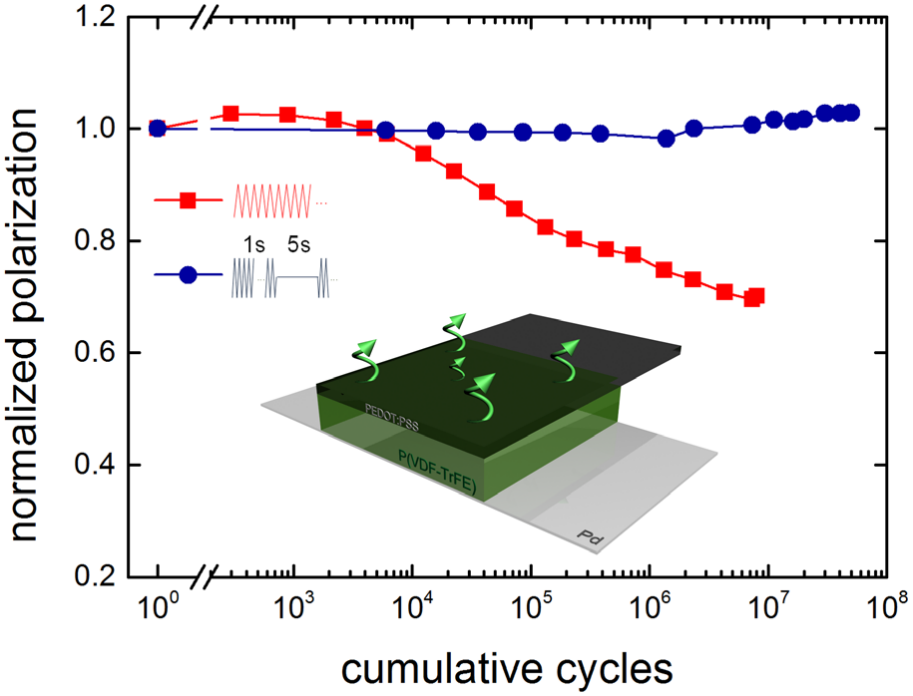
Fatigue-free P(VDF-TrFE) ferroelectric capacitors. Fatigue measurement of a P(VDF-TrFE) capacitor with a PEDOT:PSS top electrode. The red curve shows the normalized polarization as a function of the cumulative number of cycles for continuous cycling with a bipolar triangular waveform with a frequency of 100 Hz and an amplitude of 40 V. The blue curve corresponds to measurements using a waiting time of 5 seconds and a frequency of 1 kHz. The inset depicts the facilitated gas diffusion through the top polymeric electrode. A fatigue-free capacitor is realized with a programming cycle endurance of 10^8^ cycles.

Fig. 7 shows the normalized polarization as a function of the cumulative number of cycles for a PVDF-TrFE capacitor with a PEDOT:PSS top electrode. The red curve shows fatigue under continuous operation. Although improved with respect to a Au top electrode, the capacitor still shows fatigue. Optical inspection did show that the morphology of the top electrode changes. With time, micro-voids on the surface of the PEDOT:PSS electrode become clearly visible. By introducing a waiting time of 5 seconds, represented by the blue data points, a fatigue-free capacitor is realized. The inset depicts the facilitated gas diffusion through the polymeric top electrode. In this case, the morphology of the electrode does not change. However, when we apply an additional Au capping layer, the PEDOT:PSS/Au stack becomes impermeable to volatile components and the fatigue behavior resembles that of capacitors with Au-only electrodes, *c.f.* Fig. 1. Without Au, using only a PEDOT:PSS electrode and by reducing the duty cycle, there is no degradation up to 10^8^ cycles. The programming cycle endurance of the P(VDF-TrFE) capacitor approaches that of its inorganic ferroelectric counterparts.

## Summary and Conclusion

We have systematically investigated fatigue of P(VDF-TrFE) thin-film capacitors. In a capacitor with Au electrodes the coercive field remains constant but the polarization severely decreases under continuous cycling. A thermal analysis showed that thermal stress can be disregarded. To pinpoint the origin of the measured fatigue we deliberately varied the amplitude and the frequency of the applied bipolar waveform. The dependence on amplitude shows that fatigue is related to the switching of the polarization. The onset of degradation is at about 10^4^ cycles, but the degradation rate strongly increases with increasing amplitude. The frequency dependence shows that at low frequency the polarization unambiguously decreases with the number of cycles. At high frequency the capacitor is fatigue-free because the polarization does not switch.

We argue that the origin of fatigue in P(VDF-TrFE) capacitors is delamination of the Au top electrode, as can be seen from in-situ optical micrographs. The delamination is due to gases formed by phase decomposition of the P(VDF-TrFE), induced by the high internal electric fields that occur when the polarization switches. The mechanism is confirmed by inducing similar damage using high current densities in a scanning electron microscope. We show that when the gas barrier is removed and the waveform is adapted to control the gas generation and diffusion rates, a fatigue-free capacitor is realized. The P(VDF-TrFE)-based ferroelectric capacitor can be cycled more than 10^8^ times.

## Methods

The random copolymer P(VDF-TrFE) (65%–35%) was purchased from Solvay. The weight-average molecular weight, Mw, was measured with gel permeation chromatography (GPC) versus polystyrene standards and amounted to 350 kg/mol. Capacitors were fabricated on thermally oxidized silicon monitor wafers on which 50 nm thick Au bottom electrodes on a 2 nm Ti adhesion layer were photolithographically defined. Thin P(VDF-TrFE) films with a thickness of about 500 nm were spincoated at 2000 rpm from a 5 w% solution in methylethylketone. To enhance the ferroelectricity the samples were subsequently annealed in vacuum at 140°C for 2 hours. Au as a top electrode was evaporated through a shadow mask. The device area varied from 0.059 to 1.38 mm^2^. As a top electrode we also used a thin film of the amorphous conducting polymer PEDOT:PSS, a water-based suspension of poly(3,4-ethylenedioxythiophene) stabilized with poly(4-styrenesulphonic acid) (AGFA ICP 1020 (Agfa-Gevaert). To prevent dewetting, a few drops of the nonionic Zonyl FSO-100 (DuPont) fluoro-surfactant were added to the solution. The PEDOT:PSS thickness amounted to 100 nm and the conductivity amounted to 300 S/cm. Finally, the redundant PEDOT:PSS was removed by reactive ion etching using a shadow mask.

The bottom contact was grounded. Electric displacement loops versus electric field for P(VDF-TrFE) capacitors were measured using a Sawyer-Tower circuit, consisting of a Tektronix AFG3102 function generator, a LeCroy waverunner LT372 oscilloscope and a Krohn-Hite 7602 M wide-band amplifier. The ferroelectric polarization was determined by positive-up-negative-down (PUND) measurements, using a Radiant Precision Multiferroic Test System (Radiant Technologies, Inc.). A pulse of known polarity is applied to set the capacitor in a known polarization state. Subsequently, two pulses of opposite polarity are applied and the responses are recorded. The response of the first pulse contains information about the switching plus the non-switching currents whereas the second pulse gives the non-switching current of the capacitor. Subtracting the two responses gives the net switching current. Remanent polarization was determined by integration of the net switching current in time.

The fatigue measurements were performed using a bipolar triangular or sinusoidal waveform. The frequency and amplitude were varied deliberately. After a preconfigured number of cycles the remanent polarization was determined by a PUND measurement. We investigated scaling of fatigue with device area. Within device to device variations we could not identify statistically significant differences in programming cycle endurance.

Scanning electron micrographs were acquired with a Hitachi SU8000 SEM. The sample was exposed under analytical SEM working conditions, using a 10 kV acceleration voltage with an extractor working current of 20 μA, corresponding to a probe current of 1.1 nA. Micrographs were recorded intermittently during the exposure using a low voltage of 1 kV, at a probe current of ~50 pA. The top electrode of the capacitor was grounded using carbon tape.

## Author Contributions

D.Z. and M.L. fabricated the structures. D.Z., J.Ts. and I.K. performed the measurements. D.Z., I.K. and K.A. analyzed experimental results. G.G. conducted the SEM experiment. D.d.L. designed the experiment and supervised the work. D.Z., I.K., M.L., K.A., J.Ts., G.G., J.Ta., P.B. and D.d.L. co-wrote and commented on the manuscript.
